# Higher Prevalence of Thyroid Dysfunction in Type 2 Diabetes Mellitus: Effects on Glycemic Control, Diabetic Complications and Comorbidities

**DOI:** 10.3390/medicina61081427

**Published:** 2025-08-08

**Authors:** Yunus Catma, Ahmed Edizer, Osman Faruk Bayramlar, Nurdan Gul, Ozlem Soyluk Selcukbiricik, Kubilay Karsidag, Ayse Kubat Uzum

**Affiliations:** 1 Department of Internal Medicine, Istanbul Faculty of Medicine, Istanbul University, 34093 Istanbul, Turkey; 2 Department of Public Health, Bakirkoy District Health Directorate, 34140 Istanbul, Turkey; 3 Division of Endocrinology and Metabolism, Department of Internal Medicine, Istanbul Faculty of Medicine, Istanbul University, 34093 Istanbul, Turkey

**Keywords:** thyroid dysfunction, type 2 diabetes mellitus, thyroid hormones, glycemic control, diabetic complications, comorbidities

## Abstract

*Background and Objectives*: Thyroid dysfunction (TD) is more frequently observed in patients with diabetes mellitus (DM) compared to the general population. This study aims to determine the prevalence of TD in a large cohort of patients diagnosed with type 2 diabetes mellitus (T2DM) and to evaluate its possible impact on glycemic control, comorbidities, and diabetes-related complications. *Materials and Methods*: A total of 723 patients with type 2 diabetes mellitus (47.9% female, 52.1% male) were retrospectively evaluated. Demographic information of the patients, comprehensive history including onset and duration of DM and also comorbid diseases, diabetes-related complications, laboratory results, antidiabetic drugs and presence of TD were recorded and analyzed. *Results*: The prevalence of TD was 21.4% in 723 patients. Dyslipidemia was the most common comorbidity (63.6%). Patients with TD had significantly higher baseline BMI and longer diabetes duration (*p* = 0.007 and *p* = 0.048, respectively). Overall complication and comorbidity rates were 80.1% and 66%. TD was more common in females (73.4% vs. 26.6%; *p* < 0.001). Hypertension (69.5% vs. 58.7%) and neuropathy (40.9% vs. 33.0%) were significantly more frequent in the TD group (*p* < 0.05 for both). The total comorbidity rate was also higher in TD-positive patients (72.7% vs. 64.1%; *p* = 0.046). A significant positive correlation was observed between BMI and TSH levels. *Conclusions*: The increased prevalence of TD in patients with T2DM was clearly demonstrated. Female gender was identified as an independent risk factor, while elevated BMI and longer diabetes duration showed significant associations with TD status. The coexistence of TD and T2DM may contribute to a higher risk of diabetic complications and comorbidities. Routine screening of thyroid function is recommended to enable early identification and improve the overall clinical management.

## 1. Introduction

Thyroid dysfunction (TD), an endocrine disorder commonly seen in older adults and females, is more frequently observed in patients with diabetes mellitus (DM) than in the general population and may present in overt or subclinical forms of hypothyroidism or hyperthyroidism [1]. Poor glycemic control, elevated HbA1c levels, and a prolonged duration of inadequately managed DM have been associated with both increased thyroid nodule size and the development of thyroid dysfunction [2]. Moreover, the therapeutic benefits of metformin in managing TD have been demonstrated, largely due to its anti-proliferative effects in patients with DM [3].

Thyroid hormones play a crucial role in regulating glucose metabolism. Moreover, this relationship is considered bidirectional, as preexisting DM can also alter thyroid hormone profiles. Consequently, TD and DM mutually influence each other, potentially leading to adverse outcomes such as diabetic ketoacidosis. Recognizing the impact of TD on diabetic complications is therefore essential for optimizing disease management, slowing progression, and improving treatment outcomes in patients with DM [4]. Both hyperthyroidism and hypothyroidism have been shown to negatively affect metabolic control in diabetes mellitus [5].

Type 2 diabetes mellitus (T2DM) is a metabolic, multisystem disorder characterized by insulin resistance, insulin deficiency, and persistent hyperglycemia. T2DM is associated with increased morbidity and mortality, primarily due to renal failure, cardiovascular diseases (CVD), and microvascular or macrovascular complications [6,7]. While it is well established that autoimmune thyroiditis frequently coexists with type 1 diabetes mellitus (T1DM), the prevalence of thyroid dysfunction among patients with T2DM remains unclear [8].

In the present study, we aimed to provide detailed demographic and clinical information and assess the prevalence of thyroid dysfunction in a large cohort of patients diagnosed with T2DM. Additionally, we aimed to compare glycemic control, the presence of comorbidities, and diabetic complications in patients with and without TD.

## 2. Materials and Methods

### 2.1. Study Design and Participants

This retrospective cross-sectional study was conducted at the Istanbul University, Istanbul Faculty of Medicine, Department of Endocrinology and Metabolic Diseases Outpatient Clinic. We included all patients diagnosed with diabetes mellitus (DM) who were admitted to the outpatient clinic of the hospital between January 2017 and January 2020 for medical record screening. Subsequently, we retrospectively reviewed the medical records of 2164 patients, of whom 723 met the inclusion criteria and formed the final study sample.

The inclusion criteria were as follows: (1) A diagnosis of type 2 diabetes mellitus (T2DM) confirmed by an expert multidisciplinary team specializing in endocrine disorders based on international consensus criteria (HbA1c ≥ 6.5%, fasting plasma glucose ≥ 126 mg/dL, random plasma glucose ≥ 200 mg/dL in a patient with classic symptoms of hyperglycemia, or an oral glucose tolerance test with plasma glucose ≥ 200 mg/dL two hours after glucose ingestion) [9].

(2) Regular follow-up in the outpatient clinic with at least one thyroid hormone measurement recorded within the last year.

**Figure 1:**
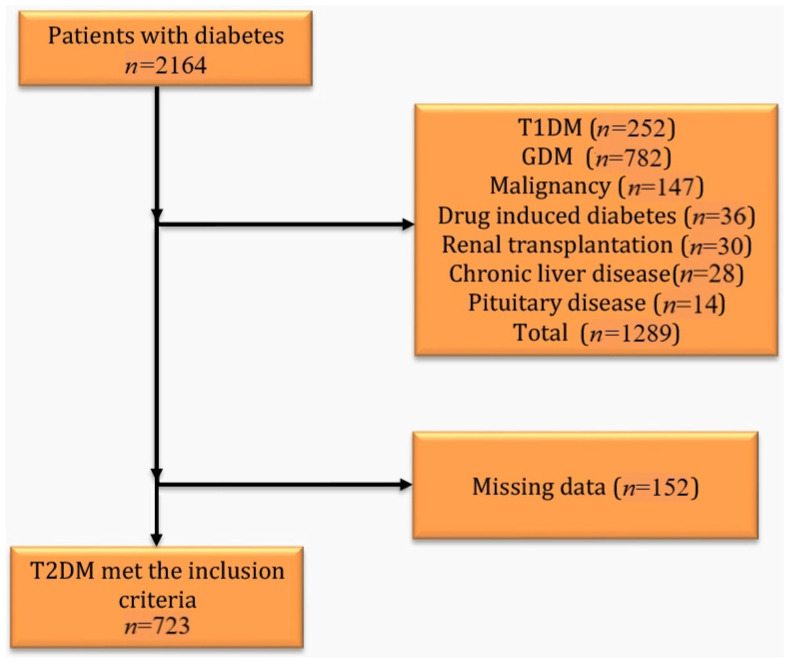
Study design: among 2164 patients with diabetes who presented to the outpatient clinic during the study period, 723 T2DM patients who met the inclusion criteria were included.

Patients with type 1 diabetes mellitus (*n* = 252), gestational diabetes mellitus (*n* = 782), malignancy (*n* = 147), drug-induced diabetes (*n* = 36), a history of renal transplantation (*n* = 30), chronic liver disease (*n* = 28), and pituitary disease (*n* = 14) were excluded from the present study. Additionally, those who did not comply with the scheduled follow-up procedures (*n* = 152) were also excluded. Detailed information on patient selection is provided in Figure 1.

We categorized the study subjects into two groups according to the presence of TD for comparative analyses. Serum thyroid function tests (TSH: Thyroid-Stimulating Hormone, fT4: Free Thyroxine, and fT3: Free Triiodothyronine) were utilized in our study as the primary criteria for thyroid dysfunction definition and classification. Thyroid dysfunction was classified into four principal categories based on laboratory reference ranges: hyperthyroidism (suppressed TSH with elevated free T4 and/or free T3), subclinical hyperthyroidism (low but measurable TSH with normal free T4 and free T3), hypothyroidism (elevated TSH with low free T4 and normal or low free T3), and subclinical hypothyroidism (elevated TSH with normal free T4 and free T3) [10]. Additionally, we also included patients who were on pharmacologic treatment for thyroid illness.

### 2.2. Demographic and Clinical Variables

Data on demographic characteristics, medical history (onset and duration of DM, family history), presence of comorbidities, physical examination findings, diabetes-related microvascular and macrovascular complications, tobacco use, alcohol consumption habits, weight, BMI, blood pressure measurements, laboratory findings, antidiabetic treatments, and the presence of thyroid dysfunction were obtained from medical records.

### 2.3. Measures

Laboratory analyses were performed on venous blood samples from the patients. Biochemical analyses were conducted using serum samples with an electrochemiluminescence immunoassay analyzer (Beckman Coulter Unicel DXI 800, Brea, CA, USA). Serum hormone levels were measured using an immunodiagnostic system (Modular E170, Roche Diagnostics, Mannheim, Germany).

HbA1c levels were analyzed with a Beckman Coulter AU480 automated HbA1c analyzer (Beckman Coulter Inc., Brea, CA, USA) using the turbidimetric immunoinhibition method. Serum TSH results were evaluated within the normal reference range of 0.27–4.2 mIU/L, free T4 within 12–22 pmol/L, and free T3 within 3.1–6.8 pmol/L.

This study was conducted with Institutional Review Board approval, dated 17 July 2020, with protocol number 29624016-050.99-1017.

### 2.4. Statistical Analysis

All data were analyzed using SPSS (Statistical Package for the Social Sciences) software for Windows (v21.0; IBM, Armonk, NY, USA). Individual and aggregate data were summarized using descriptive statistics, including means, standard deviations, medians (min-max), frequency distributions, and percentages. The normality of data distribution was assessed using the Kolmogorov–Smirnov test.

Comparisons of variables with a normal distribution were performed using Student’s *t*-test. For variables that were not normally distributed, the Mann–Whitney U test was used for group comparisons. Categorical variables were evaluated using the Chi-square test. Correlations were analyzed using Spearman’s rho or Pearson’s correlation test. A *p*-value of <0.05 was considered statistically significant.

## 3. Results

In the present study, we analyzed the data from 723 patients, of whom 346 were female (47.9%) and 377 were male (52.1%). The mean age was 58.3 ± 11.2 years (min–max: 25–82 years) and significantly higher in female patients than in male patients (59.4 ± 12.1 years vs. 57.3 ± 11.8 years; *p* = 0.017). The general clinical characteristics of the sample group are presented in Table 1.

The mean age at diabetes onset was 47.7 ± 1.5 years (min–max: 18–80 years), and the mean diabetes duration was 10.7 ± 7.9 years. The mean smoking exposure was 12.8 pack-years, which was approximately four times higher in males compared to females (19.2 vs. 5.8 pack-years). In the study group, 80.1% (*n* = 579) of the patients had at least one complication, and 66% (*n* = 477) had at least one comorbidity. The most common comorbidity was dyslipidemia, observed in 63.6% of the patients (*n* = 460), followed by hypertension (61.0%, *n* = 441), coronary artery disease (25.7%, *n* = 186), chronic kidney disease (14.1%, *n* = 102), heart failure (4.3%, *n* = 31), cerebrovascular events (2.9%, *n* = 21), and peripheral artery disease (1.0%, *n* = 7). Diabetic neuropathy was identified in 34.7% of patients, nephropathy in 32.8%, retinopathy in 31.3%, and diabetic foot in 8.6%.

We found that 109 (15.1%) of the cases had overt hypothyroidism, 31 (4.3%) had subclinical hypothyroidism, 10 (1.4%) had overt hyperthyroidism, and 4 (0.6%) had subclinical hyperthyroidism. The prevalence of hypothyroidism was significantly higher in females than in males (74.2% vs. 25.8%; *p* = 0.001). Additionally, 4.1% of the total study population was undergoing levothyroxine replacement therapy for postoperative hypothyroidism.

Baseline clinical and laboratory characteristics, as well as a comparison of laboratory findings between the sample groups, are presented in Table 2.

The mean BMI (32.08 ± 6.33 kg/m^2^ vs. 30.67 ± 6.07 kg/m^2^; *p* = 0.007) and serum TSH concentrations (4.81 ± 7.51 mIU/L vs. 2.01 ± 1.28 mIU/L; *p* = 0.000) were significantly higher in patients with TD compared to those with normal thyroid function at first admission. Conversely, serum fT3 concentration was lower in patients with TD (4.48 ± 1.32 vs. 4.63 ± 0.69 pmol/L; *p* = 0.000). Moreover, the duration of diabetes was significantly longer in patients with TD than in those without TD (11.70 ± 8.02 vs. 10.38 ± 7.82 years; *p* = 0.048) (Table 2).

We compared study parameters, diabetic complications, and comorbidities between subjects with and without TD. Female gender predominated in the TD group (73.4% vs. 26.6%; *p* = 0.000). Additionally, the presence of hypertension (69.5% vs. 58.7%; *p* = 0.015), neuropathy (40.9% vs. 33.0%; *p* = 0,015), and any comorbidity (72.7% vs. 64.1%; 0,046) was significantly higher in the TD-positive group than in the TD-negative group (Table 3).

In addition, there was statistically significant, positive correlation detected between the BMI and TSH levels (r = 0.119, *p* = 0.001). Similarly, there was statistically significant, positive correlation detected between the HbA1c and TSH levels (r = 0.108, *p* = 0.04). Additionally, there was statistically significant, positive correlation detected between the HbA1c and fT4 levels (r = 0.104, *p* = 0.05) in our sample group. No significant differences were found in the prevalence of chronic kidney disease, cerebrovascular events, peripheral artery disease, coronary artery disease, chronic heart failure, dyslipidemia, diabetic retinopathy, nephropathy, or diabetic foot. Serum TSH concentration showed a weak but positive correlation with BMI (r = 0.119, *p* = 0.001) and HbA1c (r = 0.108, *p* < 0.05).

To identify independent predictors of TD in patients with T2DM, a binary logistic regression analysis was performed, including sex, BMI, age, and diabetes duration as covariates. The overall model was statistically significant (χ^2^ = 54.06, df = 4, *p* < 0.001), indicating that the predictors reliably distinguished between TD-positive and TD-negative patients. Among the covariates, sex was the only statistically significant predictor of TD (B = −1.318, *p* < 0.001, OR = 0.268, 95% CI: 0.178–0.402), indicating that females had a significantly higher risk of developing TD compared to males. BMI (*p* = 0.337), age (*p* = 0.699), and diabetes duration (*p* = 0.584) were not significantly associated with TD status.

## 4. Discussion

It is well known that patients diagnosed with diabetes mellitus (DM) are more prone to developing thyroid dysfunction (TD) than the general population. TD appears to contribute to higher rates of comorbidities, diabetic complications, and poor glycemic control in these patients [3]. Moreover, advanced age and female sex are significant risk factors for TD development. Hypothyroidism and subclinical hypothyroidism have been reported as the most frequently occurring thyroid disorders in patients with type 2 diabetes mellitus (T2DM). In contrast, TD prevalence has been documented as 6.6% in the general adult population. The prevalence of TD among T2DM patients varies between 6% and 20% in published data [5].

Song et al. reported a prevalence of hypothyroidism at 6.8% among 1662 T2DM patients and associated older age and female sex with an increased risk of TD [11]. Similarly, Subekti et al. documented a total TD prevalence of 9.9% (7.6% hypothyroidism and 2.3% hyperthyroidism) among 303 T2DM patients [12]. On the other hand, Mehalingam et al. reported a TD prevalence of 17.5% (13.9% hypothyroidism and 3.6% hyperthyroidism) in 331 T2DM patients and noted that TD was more common in females [13]. Recent evidence by Tamali et al. similarly reported a TD prevalence of 22.1% among T2DM patients, with subclinical hypothyroidism being the most common type. They noted that longer diabetes duration, female sex, and presence of diabetic complications were significant predictors of TD [14]. Supportively, the thyroid dysfunction rate in our study population was 21.4%. Among TD patients, 15.1% were diagnosed with hypothyroidism, 2.0% with hyperthyroidism, and 4.3% with subclinical hypothyroidism. The slightly higher prevalence (21.4%) observed in our study compared to some earlier reports may be attributed to multiple factors, including regional differences in iodine intake, and genetic or metabolic variations among populations.

It is well established that iodine deficiency in different regions has a major impact on TD prevalence in the general population. Additionally, in our study, the mean age of patients with TD was 59.37 ± 12.39 years, and the proportion of female patients was significantly higher in the TD-positive group than in the TD-negative group (73.4% vs. 26.6%). The mean age was also significantly higher in female patients (59.4 ± 12.1 years) than in male patients (57.3 ± 11.8 years). Furthermore, logistic regression analysis revealed that female sex was an independent risk factor for TD in present study.

In addition to female sex and advanced age, obesity (BMI > 25 kg/m^2^) and longer diabetes duration have been well-documented risk factors for thyroid dysfunction. Hypothyroidism has been associated with increased BMI and obesity in numerous studies. Furthermore, prolonged poorly controlled DM may contribute to a more complicated clinical course of TD, as demonstrated in a study involving 354 T2DM patients and 118 non-diabetic participants, Ogbonna et al. associated DM duration > 5 years (*p* = 0.005) and central obesity (*p* = 0.001) with the presence of TD [1]. They reported significantly longer DM duration (9.5 vs. 6.0 years) in TD-positive patients compared to euthyroid patients (*p* = 0.001), concluding that DM duration > 5 years and central obesity are significant risk factors for TD. Similarly, Mehalingam et al. identified DM duration longer than 5 years as a prominent risk factor for TD in 331 T2DM patients [11]. Additionally, another study by Ogbonna et al. statistically associated higher BMI with TD development [15]. In agreement with these findings, our study showed that both BMI and duration of diabetes were significantly higher in TD-positive patients compared to euthyroid patients. This consistency supports the hypothesis that metabolic stress over time may contribute to thyroid dysfunction in T2DM.

Serum TSH levels were positively associated with hyperglycemia and insulin resistance. Increased TSH concentrations along with low free T3 and free T4 levels have been consistently reported as independent risk factors for T2DM development [5]. Furthermore, patients with T2DM often exhibit impaired TSH response and reduced peripheral conversion of T4 to T3, resulting in decreased fT3 levels [5]. Since elevated TSH with decreased fT3 and fT4 defines overt hypothyroidism, and elevated TSH with normal fT3 and fT4 defines subclinical hypothyroidism, our study found significantly higher TSH levels and lower fT3 concentrations in TD patients compared to euthyroid subjects. Ogbonna et al. reported a positive correlation between HbA1c and TSH levels in 354 T2DM patients with hypothyroidism [15]. Similarly, Elgazar et al. observed that TD prevalence was significantly higher in patients with elevated HbA1c in a cohort of 200 T2DM patients [16]. More recently, Al-Dwairi et al. demonstrated that poor glycemic control (HbA1c ≥ 7.5%) was significantly associated with insulin resistance and elevated TSH levels in T2DM population, reinforcing the link between hyperglycemia and thyroid axis disruption [17].

Numerous studies have associated TD with increased BMI and obesity. In line with these findings, our study identified a statistically significant positive correlation between BMI and TSH. Furthermore, we also observed a significant correlation between HbA1c and TSH levels, underscoring the complex interaction among adiposity, glycemic control, and thyroid function. These findings highlight the importance of an integrated endocrine approach in T2DM management, where monitoring thyroid function may provide additional insights into metabolic dysregulation.

T2DM complicated by TD may lead to adverse outcomes by increasing the risk of diabetic complications and comorbidities, particularly in poorly managed patients. Thyroid dysfunction has been significantly associated with an increased prevalence of diabetic complications and comorbidities in T2DM patients [18]. Talwalkar et al. reported that diabetic neuropathy was the most common coexisting complication among 1508 patients (including those with T2DM, hypertension, and T2DM with hypertension). Researchers highlighted that hypothyroidism prevalence in patients with T2DM, hypertension, and T2DM with hypertension was 24.8%, 33.5%, and 28.9%, respectively [19]. Similarly, Zhu et al. demonstrated that T2DM patients with TD had an increased risk of microvascular complications in a study involving 1677 T2DM patients. Researchers also noted that dyslipidemia prevalence was significantly higher in patients with TD than in those without TD [20]. In a meta-analysis reviewing 61 studies, Han et al. reported a significant association between subclinical hypothyroidism and diabetic complications, with an odds ratio (OR) of 1.74 (95% CI: 1.34–2.28) in T2DM patients [21].

In another study involving 1152 T2DM patients, Nair et al. reported a prevalence of 9.83% for clinical hypothyroidism and 5.9% for subclinical hypothyroidism. The presence of hypothyroidism was significantly associated with hypertension and dyslipidemia [22]. Similarly, in a nationwide cohort study including 18,224 T2DM cases with thyroid disease, Chen et al. documented significantly higher rates of hypertension and dyslipidemia in hypothyroid patients [23]. Furthermore, in a study of 605 T2DM patients, Zhao et al. found a significant and independent association between increased TSH levels and the development of peripheral neuropathy [24].

In accordance with published data, the most common comorbidity in our study was dyslipidemia, with a prevalence of 63.6%, followed by hypertension, with a prevalence of 61.0%. Additionally, hypertension incidence was significantly higher in the TD-positive group (69.5% vs. 58.7%) than in the TD-negative group. Similarly, neuropathy incidence was significantly higher in the TD-positive group (40.9% vs. 33.0%) compared to the TD-negative group. The total prevalence of comorbidities was also significantly increased in TD-positive patients (72.7% vs. 64.1%). This alignment between our findings and published data may be partially explained by the shared pathophysiological mechanisms linking TD with metabolic dysfunction, such as insulin resistance, endothelial impairment, and chronic low-grade inflammation. Moreover, regional variations in healthcare access, iodine status, and diagnostic thresholds may account for minor differences in prevalence rates across studies.

## 5. Conclusions

This study clearly demonstrated a higher prevalence of thyroid dysfunction (21.4%) among patients with T2DM. Logistic regression analysis revealed that female sex was an independent risk factor for TD. Although increased BMI and longer diabetes duration were not identified as independent risk factors, they were found to be significantly higher in TD-positive patients, indicating a meaningful association that warrants further investigation. Additionally, our findings suggest that T2DM patients with TD may be at increased risk for diabetic complications and comorbidities. Therefore, routine thyroid function screening may support earlier identification of at-risk individuals and improve the overall management of T2DM.

## 6. Limitations

This study was conducted as a single-center experience, which may limit the generalizability of the findings to broader populations. Additionally, some patients received thyroid hormone replacement therapy or antithyroid treatment, which may have influenced thyroid function parameters and glycemic control outcomes. It should also be considered that the prevalence of iodine deficiency varies significantly across different geographic regions, and this variable could not be assessed in our study.

Furthermore, due to the retrospective design, detailed indications for thyroid surgery and postoperative thyroid function test results were unavailable for some patients who underwent thyroidectomy. Potential confounding factors such as dietary habits, use of medications affecting thyroid function (e.g., amiodarone, corticosteroids), and autoimmune thyroid status could not be comprehensively evaluated or adjusted for in the analysis. These unmeasured variables may have influenced the observed associations and should be considered when interpreting the results.

## Figures and Tables

**Table 1:** General clinical characteristics of the study sample.

Clinical Variable	Mean ± SD	% (*n*)
The mean onset age of DM (year)	47.7 ± 1.5	-
The mean disease duration (year)	10.7 ± 7.9	-
BMI (kg/m^2^)	31.0 ± 6.2	-
Smoking exposure (packs-year)	12.8 ± 21.8	-
Family history for diabetes mellitus	-	65.1% (471)
Aged over 65 years	-	31.0% (224)
Complications		
Nephropathy	-	32.8% (237)
Retinopathy	-	31.3% (226)
Neuropathy	-	34.7% (251)
Diabetic foot (at any time)	-	8.6% (62)
Comorbidities		
Dyslipidemia	-	63.6% (460)
Hypertension	-	61.0% (441)
Chronic kidney disease	-	14.1% (102)
Cerebrovascular event	-	2.9% (21)
Coronary artery disease	-	25.7% (186)
Heart failure	-	4.3% (31)
Peripheral artery disease	-	1% (7)
Thyroid Dysfunction	-	21.4% (154)
Hyperthyroidism	-	1.4% (10)
Hypothyroidism	-	15.1% (109)
Subclinical hypothyroidism	-	4.3% (31)
Subclinical hyperthyroidism	-	0.6% (4)

BMI: Body Mass Index; DM: Diabetes Mellitus.

**Table 2:** Baseline clinical and laboratory characteristics.

	Whole Group (*n* = 723) (Mean ± SD)	TD (−) (*n* = 569) (Mean ± SD)	TD (+) (*n* = 154) (Mean ± SD)	*p*-Value
Age	58.3 ± 11.2	58.04 ± 11.87	59.37 ± 12.39	0.186
T2DM duration	10.7 ± 7.9	10.38 ± 7.82	11.70 ± 8.02	0.048 *
BMI (kg/m^2^)	31.0 ± 6.2	30.67 ± 6.07	32.08 ± 6.33	0.007 *
Glucose (mg/dL)	192 ± 82.8	195.4 ± 84.34	180.5 ± 75.84	0.063
HbA1c (%)	9.1 ± 2.4	9.20 ± 2.33	8.87 ± 2.46	0.081
TSH (mIU/L)	2.6 ± 3.6	2.01 ± 1.28	4.81 ± 7.51	0.000
fT4 (pmol/L)	16.4 ± 2.8	16.40 ± 2.27	16.62 ± 4.21	0.931
fT3 (pmol/L)	4.6 ± 0.9	4.63 ± 0.69	4.48 ± 1.32	0.000

BMI: Body Mass Index, HbA1c: Hemoglobin A1c, T2DM: Type 2 Diabetes Mellitus, TSH: Thyroid-Stimulating Hormone, fT4: Free Thyroxine, fT3: Free Triiodothyronine. * = *p* < 0.05; statistical significant. *p*-values were calculated using the Mann–Whitney U test due to non-normal distribution of the data.

**Table 3:** Comparison of study parameters, diabetic complications, and comorbidities between subjects with and without thyroid dysfunction (TD).

	Clinical Variables	Patients		*p*-Value
		TD (−) *n* (%)	TD (+) *n* (%)	
Gender	Female Male	233 (40.9%) 336 (59.1%)	113 (73.4%) 41 (26.6%)	0.000 *
Age	<65 year >65 year	398 (69.9%) 171 (30.1%)	101 (65.6%) 53 (34.4%)	0.299
Chronic kidney disease	Absent Present	489 (85.9%) 80 (14.1%)	132 (85.7%) 22 (14.3%)	0.943
Cerebrovascular event	Absent Present	553 (97.2%) 16 (2.8%)	149 (96.8%) 5 (3.2%)	0.873
Peripheral artery disease	Absent Present	563 (98.9%) 6 (1.1%)	153 (99.4%) 1 (0.6%)	0.649
Hypertension	Absent Present	235 (41.3%) 334 (58.7%)	47 (30.5%) 107 (69.5%)	0.015 *
Coronary artery disease	Absent Present	415 (72.9%) 154 (27.1%)	122 (79.2%) 32 (20.8%)	0.113
Chronic heart failure	Absent Present	548 (96.3%) 21 (3.7%)	144 (93.5%) 10 (6.5%)	0.128
Dyslipidemia	Absent Present	210 (36.9%) 359 (63.1%)	53 (34.4%) 101 (65.6%)	0.569
Retinopathy	Absent Present	394 (69.2%) 175 (30.8%)	103 (66.9%) 51 (33.1%)	0.575
Nephropathy	Absent Present	383 (67.3%) 186 (32.7%)	103 (66.9%) 51 (33.1%)	0.920
Neuropathy	Absent Present	381 (67.0%) 188 (33.0%)	91 (59.1%) 63 (40.9%)	0.043 *
Diabetic foot	Absent Present	519 (91.2%) 50 (8.8%)	142 (92.2%) 12 (7.8%)	0.696
Any complication	Absent Present	113 (19.9%) 456 (80.1%)	31 (20.1%) 123 (79.9%)	0.941
Any comorbidity	Absent Present	204 (35.9%) 365 (64.1%)	42 (27.3%) 112 (72.7%)	0.046 *

TD: Thyroid Dysfunction. * = *p* < 0.05; statistical significant. Comparisons of categorical variables were performed using the Chi-square (χ^2^) test.

## Data Availability

The original contributions presented in this study are included in the article. Further inquiries can be directed to the corresponding author.
